# L-Arginine Depletion Improves Spinal Cord Injury via Immunomodulation and Nitric Oxide Reduction

**DOI:** 10.3390/biomedicines10020205

**Published:** 2022-01-18

**Authors:** Céline Erens, Jana Van Broeckhoven, Cindy Hoeks, Gernot Schabbauer, Paul N. Cheng, Li Chen, Niels Hellings, Bieke Broux, Stefanie Lemmens, Sven Hendrix

**Affiliations:** 1 Department of Immunology and Infection, Biomedical Research Institute, Hasselt University, 3590 Diepenbeek, Belgium; celine.erens@uhasselt.be (C.E.); jana.vanbroeckhoven@uhasselt.be (J.V.B.); cindy.hoeks@uhasselt.be (C.H.); niels.hellings@uhasselt.be (N.H.); bieke.broux@uhasselt.be (B.B.); stefanie.lemmens@uhasselt.be (S.L.); 2 Institute for Vascular Biology, Center for Physiology and Pharmacology, Medical University of Vienna, A-1090 Vienna, Austria; gernot.schabbauer@meduniwien.ac.at; 3 Christian Doppler Laboratory for Arginine Metabolism in Rheumatoid Arthritis and Multiple Sclerosis, Centre of Physiology and Pharmacology, Medical University of Vienna, A-1090 Vienna, Austria; 4 Department Research and Development, Bio-Cancer Treatment International Limited, Hong Kong 999077, China; paulnmcheng@gmail.com (P.N.C.); chenli@bio-cancer.com (L.C.); 5 Institute for Translational Medicine, Medical School Hamburg, 20457 Hamburg, Germany

**Keywords:** arginase-1, CNS trauma, neuroinflammation, nitric oxide, T cells

## Abstract

Background: Spinal cord injury (SCI) elicits robust neuroinflammation that eventually exacerbates the initial damage to the spinal cord. L-arginine is critical for the responsiveness of T cells, which are important contributors to neuroinflammation after SCI. Furthermore, L-arginine is the substrate for nitric oxide (NO) production, which is a known inducer of secondary damage. Methods: To accomplish systemic L-arginine depletion, repetitive injections of recombinant arginase-1 (rArg-I) were performed. Functional recovery and histopathological parameters were analyzed. Splenic immune responses were evaluated by flow cytometry. Pro-inflammatory gene expression and nitrite concentrations were measured. Results: We show for the first time that systemic L-arginine depletion improves locomotor recovery. Flow cytometry and immunohistological analysis showed that intraspinal T-cell infiltration was reduced by 65%, and peripheral numbers of Th1 and Th17 cells were suppressed. Moreover, rArg-I treatment reduced the intraspinal NO production by 40%. Histopathological analyses revealed a 37% and 36% decrease in the number of apoptotic neurons and neuron-macrophage/microglia contacts in the spinal cord, respectively. Conclusions: Targeting detrimental T-cell responses and NO-production via rArg-I led to a reduced neuronal cell death and an improved functional recovery. These findings indicate that L-arginine depletion holds promise as a therapeutic strategy after SCI.

## 1. Introduction

Spinal cord injury (SCI) is a devastating event characterized by the permanent loss of locomotor, sensory, and autonomic functions below the level of the injury. Currently, the available clinical treatments only provide a limited improvement in patient outcomes [1].

Following SCI, a wide variety of inflammatory cells is activated and migrates towards the lesion area in a time-dependent manner [2,3]. Initially, local cells (e.g., microglia and astrocytes) are predominant, yet over the course of days, peripheral immune cell infiltration occurs [3,4,5,6]. Lymphocytes accumulate in the spinal cord days after trauma and remain chronically present [2,3]. The role of different T cell subpopulations in SCI, especially of autoreactive T cells, has been intensely debated during the last decades [7,8]. Some researchers suggested that the presence of autoreactive T cells is beneficial and promotes functional recovery [9,10,11]. Others have reported that the transfer of autoimmune T cells exacerbated the neuropathology after SCI [12]. It has been speculated that the opposing results may depend on the lymphocyte polarization status [11,13,14]. Moreover, several studies demonstrated a general improvement after complete ablation of T-cell responses [15,16,17,18]. 

L-arginine is a semi-essential amino acid and a precursor for multiple processes, including the production of polyamines, collagen, and nitric oxide (NO) [19]. Its availability in pathological conditions is mainly regulated by the expression of two enzymes; nitric oxide synthase (NOS) and arginase-1 (arg-1). In SCI, NOS expression is significantly upregulated. The associated NO production in macrophages and microglia is seen as a mediator for the occurring secondary damage [20]. The administration of NOS inhibitors and the observed improvements after SCI by several research groups confirmed the detrimental role of NOS [21,22].

Furthermore, activated T cells rapidly metabolize L-arginine. The degradation of L-arginine via arg-1 by myeloid-derived suppressor cells (MDSCs) is described to impair T-cell responsiveness [23,24,25]. In line with this notion, administration of arg-1 was found to attenuate and delay the onset of experimental autoimmune encephalomyelitis (EAE), reduce the T helper 1 (Th1) and T helper 17 (Th17) percentage, and decrease the associated concentration of pro-inflammatory cytokines (e.g., IL-17, IFN-Ὑ) in vitro [26]. 

L-arginine is increased in the local microenvironment after SCI. However, it is unclear whether it plays a protective or degenerative role [27,28]. Supplementation of L-arginine after SCI resulted in beneficial effects in some studies and detrimental effects in others [29,30,31,32]. To date, the role of L-arginine in the traumatic inflammatory processes after SCI, still remains to be clarified.

In the present study, we investigated the effect of L-arginine depletion in the context of SCI-induced inflammation via administration of recombinant human pegylated arginase-1 (rArg-I), a compound extensively studied for cancer treatment [33]. We are the first to show that functional recovery is improved by repeated injections of rArg-I. Systemic depletion of L-arginine reduced focal CD4^+^ T-cell infiltration and acute splenic expansion of Th1 and Th17 cells in vivo. Furthermore, decreased NO production and neuronal cell death were found in the spinal cord, suggesting reduced NO-mediated neurotoxicity. These data highlight the importance of T-cell immunomodulation and diminishing the NO production via L-arginine depletion with rArg-I for recovery after SCI.

## 2. Materials and Methods

### 2.1. Animals and Human Samples 

All animal experiments were performed using 9- to 11-week-old female wild-type CD57BL6/j mice obtained from Janvier Labs (France). Mice were housed in groups in the conventional animal facility at Hasselt University under standardized conditions on a 12 h light-dark cycle and in a temperature-controlled room (20 ± 3 °C). Food and water were available ad libitum. Animal experiments were approved by the Hasselt University Ethics Committee and performed in compliance with the guidelines of the European Directive 2010/63/EU on the protection of animals used for scientific purposes. 

Human peripheral blood samples were collected from seven healthy controls (24–40 years old, six female/one male) and recruited via the University Biobank Limburg (UBiLim), according to standard procedures for recruitment of heathy donors. Briefly, blood donors from the Biomedical research institute were contacted and asked to sign an informed consent prior to donation. Their health status was confirmed via a questionnaire. Blood was collected by a trained physician, and samples were coded to comply with the privacy legislation and ensure data protection. This study was approved by the local medical ethical committee of Hasselt University, and informed consent was obtained from all donors (Approval codes 201712, 201760, 201829, and UH-IMMMAC-P1 accepted on 23 February 2017, 9 November 2017, 19 November 2018, and 18 October 2019, respectively).

### 2.2. Experimental Spinal Cord Injury and Recombinant Arginase-I Treatment

SCI by means of a T-cut hemisection was performed by a standard operating procedure as previously described [34,35]. In brief, surgical anesthesia was induced with 3% isoflurane (IsofFlo, Abbot Animal Health) after which mice underwent a partial laminectomy at thoracic level eight (T8). Using iridectomy scissors, a bilateral hemisection was induced, transecting the dorsomedial and ventral corticospinal tract. The muscles were sutured, and the skin was closed using wound clips (Autoclip, Clay-Adams Co., Inc., Becton-Dickinson, Erembodegem, Belgium). Mice were randomly operated (either vehicle or treatment group), and investigators remained blind to these groups for the duration of the study. Bladders of the mice were emptied manually daily until the micturition reflex was restored spontaneously. To control the quality of the T-cut hemisection, all spinal cords were checked for excessive bleeding, atopical contusions (bruising), standardized depth and breadth of the cut as well as the absence of bone fragments and hairs intra-operatively. After the operation, abnormal BMS scores serve as further quality control of the T-cut hemisection (see below, ‘Section 2.3′, exclusion criteria).

**Figure 1:**
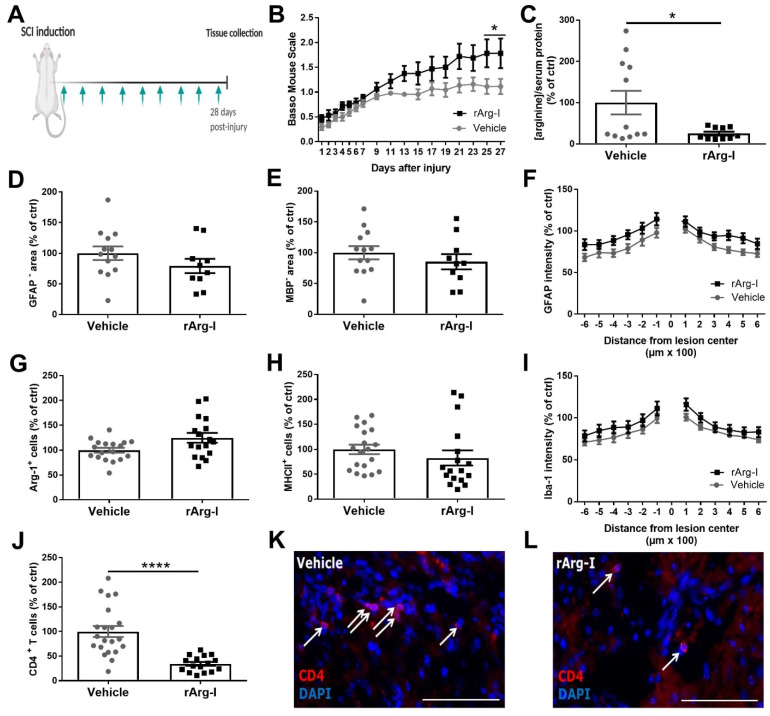
rArg-I treatment improves recovery and reduces intraspinal CD4^+^ T cell numbers 28 days after trauma. (**A**) Schematic representation of the treatment protocol. A T-cut hemisection was performed, and rArg-I (50 mg/kg) was injected intraperitoneally every 3 days starting at the day of injury, indicated by the green arrows. Mice were sacrificed 28 days post-injury (dpi). (**B**) Treatment with rArg-I improved functional recovery after spinal cord injury (SCI). Recovery of hind limb motor function was determined using the Basso Mouse Scale. *n* = 16–22 mice/group. [F(1,36) = 4.932, *p* = 0.0115, two-way ANOVA]. (**C**) Serum arginine depletion was found after rArg-I treatment 28 dpi. *n* = 10–12 mice/group. [100 ± 28.56 vs. 25.29 ± 4.55, *p* = 0.0287, two-tailed unpaired student t-test]. (**D**–**J**) Histological analyses of spinal cord sections 28 dpi. (**D**) Lesion size, (**E**) demyelinated area, (**F**) astrogliosis, (**G**,**H**) number of arg-1^+^ and MHCII+ cells, and (**I**) Iba-1 intensity did not change between treatment groups. *n* = 10–19 mice/group. (**J**) The number of infiltrating CD4^+^ T cells was significantly reduced in the spinal cord of mice treated with rArg-I compared to vehicle control 28 dpi. *n* = 16–21 mice/group. [100 ± 11.09 vs. 34.33 ± 3.98, *p* < 0.0001, two-tailed unpaired student t-test]. Representative images of CD4^+^ T cells (white arrows) in (**K**) vehicle- and (**L**) rArg-I-treated mice, respectively. Scale bars represent 75 µm. Data are pooled from two independently performed experiments and represent mean ± SEM. * *p* < 0.05 and **** *p* < 0.0001.

In experiments regarding systemic L-arginine depletion, mice were injected intraperitoneally (50 mg/kg) every 3 days with rArg-I starting 4 h post-surgery (Figure 1A). The control group was treated according to the same treatment protocol and received equal amounts of vehicle solution (Na_2_HPO_4_ and NaH_2_PO_4_, pH 7.4).

rArg-I was produced in the laboratory of Prof. Dr. Cheng as follows: recombinant human arginase was attained by producing the His-tagged human arg-1 (liver arginase) enzyme in *B. subtilis* [36]. The arginase activity was assessed with a coupled spectrophotometric assay as described by Ikemoto et al. [37]. The purified enzyme has a specific activity of ±400 IU/mg protein. One international unit of arginase is defined as the amount of enzyme that can produce 1 µmol urea/min at 30 °C, pH 8.5. Next, mPEF-SPA of MW 5000 was covalently attached to purified recombinant human arginase according to the same methods used for formulating ADI with PEG [38]. The resulting PEG-formulated (pegylated) arg-1 was termed rhArg-peg5,000 mw (rArg-I). This enzyme enables L-arginine depletion via the metabolism of L-arginine. Arg-1 hydrolyses L-arginine to ornithine and urea [39].

### 2.3. Assessment of Locomotor Recovery after Spinal Cord Injury

Locomotor recovery after injury was measured starting 1 day post-injury (dpi) over a period of 4 weeks according to the Basso Mouse Scale (BMS) [40]. This 10-point certified scale ranges from zero to nine, indicating complete hind limb paralysis or normal locomotion, respectively. The hind limb movements were evaluated in a blinded manner in an open field during a 4-min interval. During the first week, mice were scored daily and thereafter every other day till the end of the experiment (28 dpi). The average of the left and right hind limb scores for each animal was used for analysis. Mice were excluded from the experiment under the following conditions: (i) an average BMS score of zero at the end of the observation period (as an indication that the lesion was abnormally severe), or (ii) an average BMS score of two immediately after SCI (as an indication that the T-cut hemisection was incomplete) to guarantee the standardization within each experiment according to lesion severity. To evaluate functional and histopathological recovery after rArg-I treatment, 44 animals were initially in the experiment. Two mice died after surgery, four mice were excluded based on the exclusion criteria described above (vehicle *n* = 22, rArg-I *n* = 16).

### 2.4. Plasma Preparation and L-Arginine ELISA

The systemic L-arginine concentration was determined 4, 7, 12, or 28 dpi. Mice received an overdose of Nembutal, and blood was transcardially collected. The samples were kept on ice in EDTA and centrifuged (1000× *g*, 15 min, 4 °C) to remove cellular debris and platelets. The remaining supernatant was stored at −20 °C and used for analysis. The systemic L-arginine concentration was quantified utilizing the L-arginine ELISA (K7733, Immundiagnostik, Bensheim, Germany) according to the manufacturer’s protocol. The absorption was determined with an ELISA plate reader (iMarkTM microplate reader, Bio-Rad, Temse, Belgium) at 450 nm and 655 nm. The obtained results were corrected for the total protein plasma levels established using the BCA Protein Assay Kit (Thermo Fisher Scientific, Brussel, Belgium).

### 2.5. Immunohistochemistry

Mice were transcardially perfused with Ringer solution containing heparin, followed by perfusion with 4% paraformaldehyde in phosphate buffered saline (PBS, pH 7.4). The spinal cord was isolated 28 dpi, and 10 µm thick serial sagittal cryosections were made. For immunohistochemical analysis, the cryosections were pre-incubated with 10% protein block (X0909, Dako, Heverlee, Belgium) supplemented with 0.1% Triton X-100 for 30 min at room temperature (RT). Primary antibodies (Supplementary Table S1) were incubated overnight at 4 °C in PBS with 1% protein block and 0.05% Triton X-100. After repeated washing steps with PBS, secondary antibodies were incubated for 1 h at RT (Supplementary Table S2). Tissue sections were counterstained with DAPI and mounted with fluorescence anti-fade mounting medium (S3023, Dako). Images from five to six sections per animal were taken with a Leica DM 4000 B LED Fluorescence microscope coupled to the Leica DFC 450C digital sight camera (Leica, Machelen, Belgium) for further analysis.

### 2.6. Quantitative Image Analysis

Quantitative image analysis was performed on unmodified photos by an investigator blinded to the treatment groups as previously described [34,35]. For standardization, five to six spinal cord sections per mouse were quantified using the ImageJ open source software version 1.51j8 (National Institutes of Health, Bethesda, MD, USA). Lesion size and demyelination were evaluated by studying the lesion epicenter and delineating the glial fibrillary acidic protein (GFAP) and myelin basic protein (MBP) negative area, respectively. Astrogliosis and microglia/macrophage infiltration was examined via intensity analysis in the perilesional area of GFAP and ionized calcium-binding adaptor molecule (Iba-1). Th cells were identified as cluster of differentiation 4 (CD4) positive and Iba-1 negative cells and counted throughout entire spinal cord sections. Microglia/macrophage activation was quantified by manually counting the perilesional arg-1 and major histocompatibility complex II (MHCII) positive cells. Quantification of microglia/macrophage and axon interactions was conducted by counting the number of neurofilament (NF) positive dystrophic axon bulb and DAPI^+^Iba-1^+^ cell contacts. Dystrophic axon bulbs were identified based on their globular morphology at the end of an axon. Analysis was performed at the perilesional area. Lastly, neuronal cell death was studied by evaluating cleaved caspase 3 positive and neuronal nuclei (NeuN) positive cells. The number of cleaved caspase 3 positive and NeuN positive cells were both quantified. Double positive cells were normalized over the total number of NeuN^+^ cells to adjust for differences in neuronal cell count between pictures. All quantifications were normalized to the control group. To maximize readability of the figures, the contrast and brightness of the images were enhanced to the same extent in all groups. 

### 2.7. Splenocyte and Peripheral Blood Mononuclear Cell Isolation

Primary murine splenocytes were isolated from naive or SCI mice at the indicated time points after injury. The spleens were surgically removed and mashed through a 70 µm cell strainer. The obtained single-cell suspensions were subjected to red blood cell lysis by an incubation step in 0.83% ammonium chloride for 4 min at RT. Entire splenocyte mixtures were used for phenotypical characterization of the systemic immune response or to study the effect of rArg-I on T-cell proliferation ex vivo. Human peripheral blood mononuclear cells (PBMCs) were isolated from peripheral blood employing a Ficoll gradient.

### 2.8. T-Cell Characterization after Spinal Cord Injury

Splenocytes were isolated 4, 7, and 12 dpi and analyzed by flow cytometry directly ex vivo. Cells were stained with anti-mouse CD3 FITC (100203), CD11b PerCP/Cyanine5.5 (101228), CD183 PE (126505), CD196 PE/Cyanine7 (129815), CD25 APC (102011), CD45 Alexa Fluor 700 (103127), CD4 Pacific Blue (100427), CD8 Brilliant Violet 510 (100751), CD19 Brilliant Violet 650 (115541), Ly6C Brilliant Violet 785 (128041) and Zombie NIR (423105, all from BioLegend, Amsterdam, The Netherlands). After staining, cells were acquired on a BD LSRFortessa instrument using FACSDiva software (both BD Biosciences, Erembodegem, Belgium). Manual gating, FlowAI, and statistics were performed using FlowJo V.10.7.1 software (FlowJo LLC., Ashland, OR, USA) and Graphpad Prism version 7.04 software (Supplementary Figure S1). Unbiased clustering of data was performed using OMIQ (Omiq, Inc). Manual gating of single, live, CD45^+^ leukocytes was performed, after which 10 × 10^5^ cells were subsampled for each group (dpi + treatment). FlowSOM was run on pooled data, generating 49 clusters and 10 metaclusters. These metaclusters were overlayed on a UMAP plot of the same data, and a clustered heatmap was generated, showing scaled MFI data for each marker from low (blue) to high (red) expression.

### 2.9. T-Cell Proliferation Assay

Splenocytes from naive mice were labeled with the Cell Trace Yellow Cell Proliferation Kit (C34567, Thermo Fisher Scientific). The labeled cells were subsequently seeded in 96-well U-bottom plates at 125 000 cells/well in RPMI-1640 medium (Lonza, Bornem, Belgium) supplemented with 10% fetal bovine serum (FBS, Gibco). Mouse T cells were activated with soluble anti-CD3ϵ (2 µg/mL, 40-0031, Tonbo Biosciences) and anti-CD28 (1 µg/mL, 40-0281, Tonbo Biosciences) and incubated with the indicated concentrations of rArg-I or vehicle solution for 120 h at 37 °C with 5% CO_2_. Prior to acquisition, cells were stained with Zombie NIR, anti-mouse CD3 FITC, CD4 Pacific Blue, and CD8 Brilliant Violet 510 (all Bioledgend).

Human PBMCs were stained with CellTrace Violet Cell proliferation kit (C34571, Thermo Fisher Scientific) and seeded at a density of 2 × 10^5^ cells/well in RPMI-1640 medium (Lonza) supplemented with 10% FBS in a 96-well U-bottom plate. Cells were activated with Treg suppression inspector beads (1:1, 130-092-909, Miltenyi Biotec, Leiden, The Netherlands) and supplemented with the indicated concentrations of rArg-I or vehicle solution for 6 days at 37 °C/5% CO_2_. Prior to acquisition, PBMCs were stained with fixable viability dye eFluor 506 (65-0866-14, Thermo Fisher Scientific), mouse anti-human CD4 APCeFluor 780 (47-0048-42, Thermo Fisher Scientific) and mouse anti-human CD8 PerCP (564526, BD Biosciences). As a quality control, samples were excluded if the percentage of proliferation for the vehicle solution remained below 40%. Cells were acquired using the BD FACS-Fortessa and T-cell proliferation was analyzed using FlowJo v10.7.1. Unstimulated cells that received neither vehicle nor rArg-I treatment were used as controls to set proliferation gates.

### 2.10. Quantitative PCR

RNA from control and rArg-I-treated mice was isolated from the spinal cord 7 dpi with the RNeasy Mini Plus Kit (74136, Qiagen, Venlo, The Netherlands) as previously described [35]. A standardized area of the spinal cord tissue was collected (spanning 5 mm rostral to 5 mm caudal to the lesion center) and snap-frozen in liquid nitrogen for this purpose. In turn, RNA was reverse transcribed to cDNA using qScript cDNA Supermix (95048 –100, Quanta Biosciences). A quantitative PCR was conducted on a StepOnePlus detection system (Applied Biosystems) using universal cycling conditions. Data were normalized towards the two most stable reference genes found by geNorm. A list of the primer sequences can be found in Supplementary Table S3.

### 2.11. Western Blotting

Western blot analysis was performed to characterize macrophage polarization, as previously described [34]. Macrophages were lysed in reducing sample buffer (RIPA with protease inhibitors). The total protein concentrations were determined by means of the BCA Protein Assay Kit (23227, Thermo Scientific). Goat anti-mouse arg-1 (1/1000, sc-271430, Santa Cruz, Heidelberg, Germany) and mouse anti-mouse iNOS (1/500, N6657, Sigma-Aldrich, Bornem, Belgium) was incubated overnight at 4 °C. As a loading control, β-actin (1/2000, 47778, Santa Cruz) was used on the same blot. The PierceTM ECL Plus detection kit (32132, Thermo Scientific) was utilized to assess chemiluminescent signal detection. Images were taken with the AmershamTM Imager 680 (General Electric Company) and used for densitometric analysis with Image Quant TL software.

### 2.12. Microglial Cell Isolation

Cortical microglia were isolated from postnatal P0-P3 C57BL6/J pups as described by Tamashiro et al. [41]. Meninges and hippocampal tissue were removed where after the tissue was disrupted in Dulbecco’s Modified Eagle Medium (DMEM) with 100 µg/mL DNase using a 1 mL pipet. Afterward, the homogenate was filtered through a 70 µm cell strainer and seeded in a T75 culture flask. The cell suspension was cultured in DMEM with 10% FBS at 37 °C with 5% CO_2_ in a humidified incubator. When 80% confluency was reached, DMEM with 10% FBS and 30% L929 conditioned medium (LCM) was added. Microglia were isolated one week later by shake-off (200 rpm, 2 h, 37 °C).

### 2.13. Isolation and Culture of Bone Marrow-Derived Macrophages

Bone marrow-derived macrophages were obtained from the femur and tibia of healthy female wild-type CD57BL6/j mice. Bone marrow isolation was performed as previously described [42]. Primary cell suspensions were cultured in RPMI-1640 (BE12-115F/U1, Lonza) supplemented with 10% FBS, 50 U/mL penicillin, 50 U/mL streptomycin, and 15% LCM. Macrophage differentiation was allowed for 7 days at 37 °C and 5% CO_2_. Thereafter, cells were seeded for in vitro experiments.

### 2.14. Griess Assay

Nitrite concentrations in conditioned medium of primary microglia and macrophages were defined by the Griess reagent system kit (G2930, Promega, Leiden, The Netherlands). 75,000 cells/cm^2^ were seeded in 24-well plates and incubated with lipopolysaccharide (LPS; 0.2 µg/mL, L4524, Sigma-Aldrich), recombinant interleukin-13 (rIL-13, 33 ng/mL, 210-13, Peprotech, London, UK) or left untreated. rArg-I or vehicle solution was added in the indicated concentrations simultaneously. Medium was collected 24 h after the onset of the experiment.

Reduced local NO levels are an indication of reduced local L-arginine levels [43]. Therefore, as an indirect method to detect intraspinal L-arginine depletion in vivo, we verified the in vivo nitrite production 7 dpi, at the peak of iNOS expression [44], on snap-frozen spinal cord tissues (from 3 mm rostral to 3 mm caudal to the lesion center) with the Griess Reagent kit (ab234044, Abcam, Cambridge, UK) according to the fabricant’s protocol. The outcome of the Griess assay was normalized to the sample mass.

### 2.15. MTT Viability Assay

The MTT viability assay was performed on primary macrophages 48 h after rArg-I or vehicle incubation. Cells were also exposed to IL-13 or LPS simultaneously. Following media removal, thiazolyl blue tetrazolium bromide (MTT; 500 µg/mL, M2128, Sigma-Aldrich) was incubated for 4 h at 37 °C. Thereafter, media were removed, and formazan crystals were dissolved with DMSO. Absorbance was measured at 540 nm with an ELISA plate reader. The results were calculated as a percentage of relative MTT reduction compared to the control wells.

### 2.16. Experimental Design and Statistical Analysis

Sample sizes for animal studies were determined by power calculations for the primary parameter based on pilot studies or literature (power = 80%, α = 0.05). Data were statistically analyzed using GraphPad Prism software (GraphPad Software, Inc., La Jolla, CA, USA). The D’Agostino and Pearson omnibus normality test was used to test normal distribution. Functional recovery in vivo and histological analysis of Iba-1 intensity and astrogliosis were statistically analyzed with a two-way ANOVA for repeated measurements followed by a Bonferroni post-hoc test for multiple comparisons. The nonparametric Mann–Whitney U test was applied to evaluate differences between two groups. A two-tailed unpaired student t-test was used if the data passed the normality test. For data sets with three groups or more, a one-way ANOVA with a Dunn’s multiple comparisons test was performed. An alpha value of 5% was considered as criterion for significance. Data were reported as mean ± standard error to the mean (SEM) unless stated otherwise and considered statistically significant when *p* < 0.05. * *p* < 0.05, ** *p* < 0.01, *** *p* <0.001 and **** *p* < 0.0001. The performed statistical test, their probability (*p*-value), and the sample size are provided in the figure legends. The datasets supporting the conclusions of this article are included within the article (and its additional file(s)) and are available from the corresponding author, upon reasonable request. 

## 3. Results

### 3.1. Systemic L-Arginine Depletion via rArg-I Administration Results in Improved Functional Recovery and Strongly Reduced CD4^+^ T-Cell Numbers after Spinal Cord Injury

Due to the important role of L-arginine in inflammation, we investigated whether L-arginine depletion via rArg-I treatment improves the functional and histopathological outcome after SCI. Therefore, wild-type C57BL6/J mice underwent a T-cut hemisection followed by repeated rArg-I or vehicle treatment (Figure 1A). In both groups, mice experienced complete hind limb paralysis (BMS score zero) that gradually improved over time (Figure 1B). Mice subjected to rArg-I therapy showed a statistical enhancement of functional recovery compared to the vehicle group at the end of the 28-day observation period (Figure 1B). At this time point, a significant reduction in the serum L-arginine concentration was apparent, confirming the treatment efficacy (Figure 1C).

Histological analysis demonstrated no difference in lesion size, demyelinated area, astrogliosis, and microglia/macrophage polarization and accumulation between treatment groups (Figure 1D–I). However, the number of CD4^+^ T cells was significantly decreased to 35% in mice that received rArg-I treatment (Figure 1J–L).

### 3.2. rArg-I Treatment Alters the Systemic Immune Response after Spinal Cord Injury

**Figure 2:**
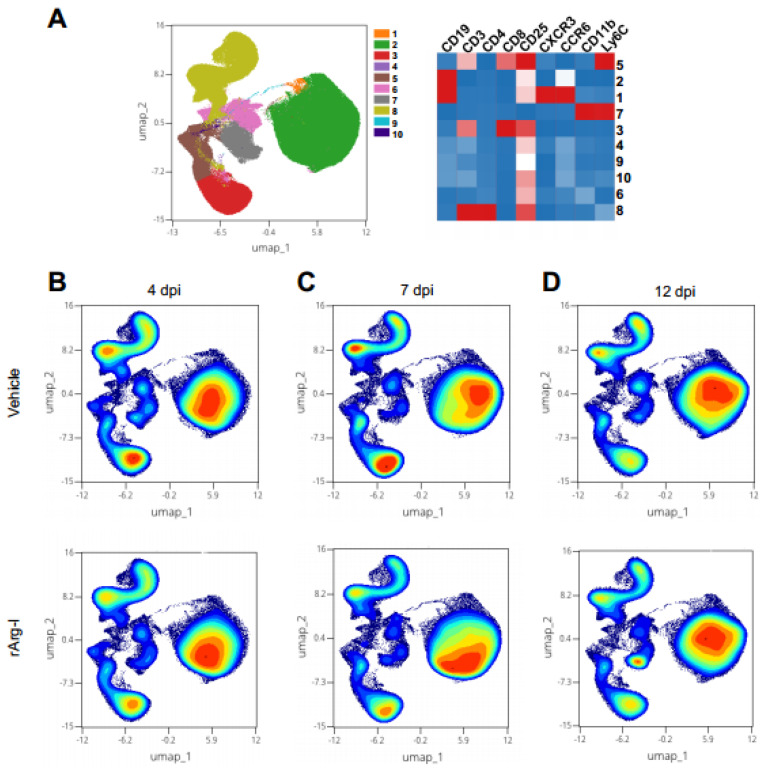
The splenic immune subsets remain unaltered within the first two weeks after spinal cord injury. (**A**) The UMAP plot, overlayed with FlowSOM metaclusters, revealed major immune subsets in the spleen, including CD4^+^ helper and CD8^+^ cytotoxic T cells, B cells, patrolling and inflammatory monocytes (**left**). The marker expression (red = high expression, blue = low expression) of these cells is indicated on the heat map (**right**). The identified cell populations are numbered from 1–10 in the UMAP and heat map, respectively. There were no major shifts in these subsets (**B**) 4, (**C**) 7, and (**D**) 12 dpi identified. Representative plots are shown. *n* = 18–20 mice/group.

**Figure 3:**
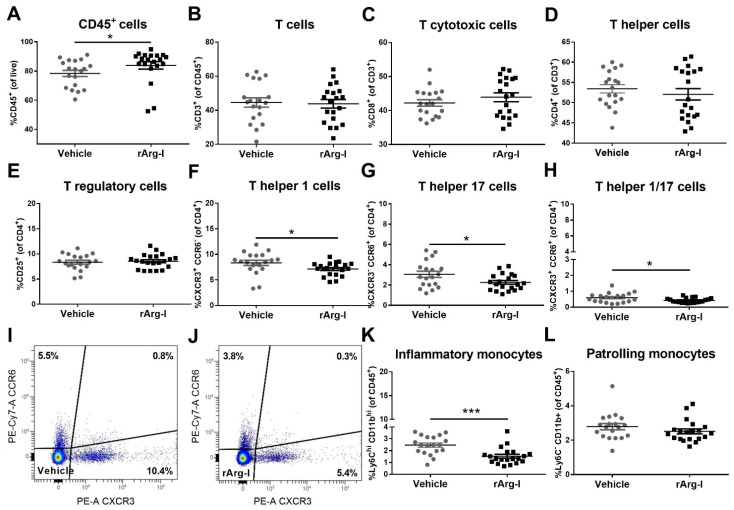
The acute splenic immune response is affected by rArg-I-mediated L-arginine depletion after spinal cord injury. (**A**) Flow cytometry indicated increased numbers of CD45^+^ leukocytes 4 dpi when mice were treated with rArg-I. *n* = 19–20 mice/group. [81.3 vs. 88, *p* = 0.0115, two-tailed Mann–Whitney U test]. L-arginine depletion had no effect on the number of splenic (**B**) CD3^+^ T cells, (**C**) CD8^+^ T cytotoxic cells, (**D**) CD4^+^ T helper cells, and (**E**) CD4^+^CD25^+^ double positive T regulatory cells 4 dpi. *n* = 19–20 mice/group. (**F**–**H**) The number of Th1, Th17, and Th1/17 cells was significantly decreased when rArg-I was applied compared to the vehicle. *n* = 19–20 mice/group. [*p* = 0.0431, *p* = 0.0253, and *p* = 0.0322, two-tailed unpaired student t-test]. (**I**,**J**) Representative dot plots of splenocytes isolated from (**I**) vehicle-treated and (**J**) rArg-I-treated mice. (**K**) rArg-I treatment significantly decreased the percentage of splenic inflammatory monocytes. *n* = 5–20 mice/group. [2.46 vs. 1.32, *p* = 0.0003, two-tailed Mann–Whitney U test]. (**L**) The number of patrolling monocytes remained unaltered 4 dpi. Data are shown as mean ± SEM. * *p* < 0.05 and *** *p* < 0.001.

T-cell functionality and NO production are strongly dependent on L-arginine availability and play an essential role in secondary inflammation after SCI [20,25,45,46]. To define whether systemic rArg-I treatment alters the peripheral immune response in the spleen after SCI, flow cytometry was performed on splenocytes at 4, 7, and 12 dpi. The UMAP plot, overlayed with FlowSOM metaclusters, show the major immune subsets in the spleen, including CD4^+^ helper and CD8^+^ T cytotoxic cells, B cells, as well as patrolling and inflammatory monocytes (Figure 2A). In general, no major shifts in these immune subsets were noted over time (Figure 2B–D). Our data show that rArg-I therapy influences the T-cell inflammatory response acutely at 4 dpi (Figure 3). A significant decrease of the percentage of Th1, Th17, and Th1/17 cells was noted in the rArg-I-treated mice (Figure 3F–J), although the total percentage of T cells, cytotoxic T cells, the total Th subset, and the regulatory T (Treg) cells remained unaltered between treatment and control groups (Figure 3B–E). Moreover, a reduction in the percentage of inflammatory monocytes was apparent when the mice were treated with rArg-I, whereas no differences were detected in the patrolling monocytes (Figure 3K,L).

The impact of rArg-I administration on the splenic T-cell response was time-dependent. The effects on Th1 and Th17 subsets disappeared 7 dpi (Supplementary Figure S2F–H). However, a reduced percentage of CD45^+^ leukocytes and CD3^+^ T cells was found at that time point (Supplementary Figure S2A,B). Treatment also influenced the percentage of patrolling and inflammatory monocytes 7 dpi (Supplementary Figure S2I,J). The percentage of CD3^+^ T cells was restored to control levels 12 dpi (Supplementary Figure S3B). Here, the overall observed effects of rArg-I on the splenic T-cell response disappeared, only the number of CD8^+^ T cytotoxic cells rose (Supplementary Figure S3C–H). Similar findings were obtained after studying the splenic phagocytes (Supplementary Figure S3I,J). The difference in percentage of inflammatory monocytes between groups disappeared, only the percentage of the patrolling monocytes remained reduced 12 dpi (Supplementary Figure S3J). All observations were made in the presence of a significantly decreased serum L-arginine concentration due to rArg-I treatment (Supplementary Figure S4).

**Figure 4:**
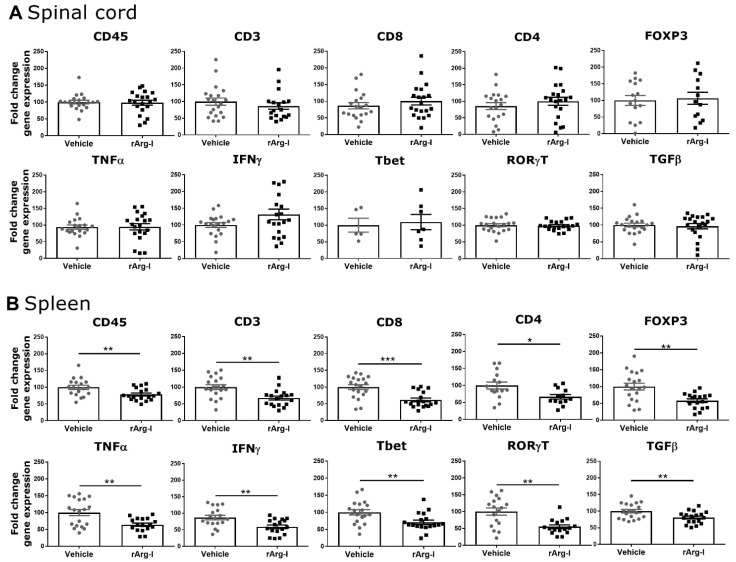
rArg-I treatment decreases the transcription of selected inflammatory genes in the spleen (systemic inflammation). (**A**) Quantitative RT-PCR analysis of spinal cord tissue isolated 7 days after spinal cord injury (SCI) induction. The expression of all the tested inflammatory genes did not change between treatment groups. *n* = 5–20 mice/group. (**B**) Quantitative RT-PCR analysis of spleen samples isolated 7 days after SCI. All analyzed inflammatory genes decreased in the rArg-I treatment group compared to vehicle control. *n* = 13–19 mice/group. [*p* < 0.05, *p* < 0.01, *p* < 0.001, two-tailed unpaired student t-test and two-tailed Mann–Whitney U test]. Data are shown as mean ± SEM, fold change of vehicle control. * *p* < 0.05, ** *p* < 0.01 and *** *p* < 0.001.

Alterations of the immune response in the spinal cord (Figure 4A) and in the spleen (Figure 4B) were investigated utilizing qPCR. No significant changes were observed for the different inflammatory markers in the spinal cord (Figure 4A). In concordance with the flow cytometry data (Figure 3 and Supplementary Figure S2), the splenic immune response was significantly altered by rArg-I treatment. A significant transcriptional reduction was detected for all the tested markers (Figure 4B). Together these findings highlight that L-arginine depletion via rArg-I treatment suppresses the acute splenic immune response.

### 3.3. L-Arginine Is Essential for T-Cell Activation

**Figure 5:**
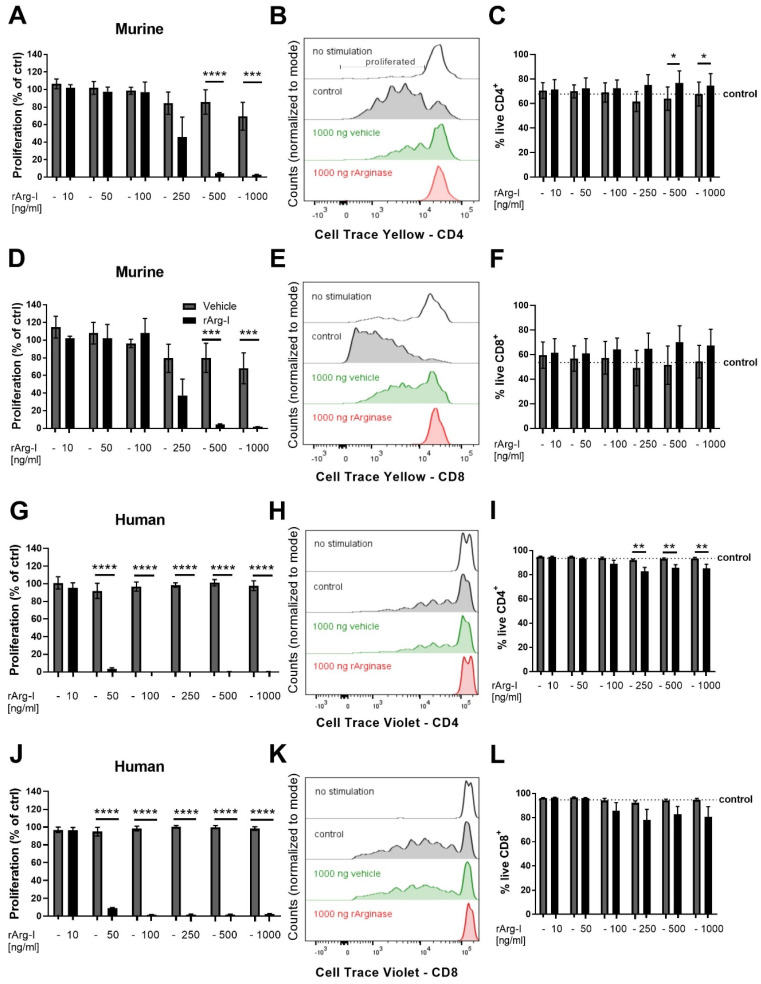
CD4^+^ and CD8^+^ murine and human T-cell proliferation is significantly reduced by rArg-I. Doubling rates of murine splenocytes and human peripheral blood mononuclear cells (PBMCs) were analyzed utilizing Cell Trace Yellow and Violet labelling, respectively. Cell viability was checked 5–6 days after the onset of the experiment. (**A**,**D**,**G**,**J**) The proliferation of murine and human T cells are indicated as percentage of control (no vehicle or rArg-I incubation). (**B**,**E**,**H**,**K**) Corresponding representative histograms. (**A**,**B**) Murine T-cell activation resulted in CD4^+^ T-cell proliferation. rArg-I addition in concentrations of 500 ng/mL or more, reduced proliferation. *n* = 5 biological repeats. [F(1,21) = 37.65, *p* < 0.0001, two-way ANOVA]. (**D**,**E**) Supplementation of rArg-I suppressed murine CD8^+^ T-cell proliferation. *n* = 5 biological repeats. [F(1,21) = 28.06, *p* < 0.0001, two-way ANOVA]. (**G**,**H**) CD4^+^ human PBMC proliferation was ablated when rArg-I concentrations equal to or higher than 50 ng/mL were added. *n* = 5–7 biological repeats. [F(1,30) = 1356, *p* < 0.0001, two-way ANOVA]. (**J**,**K**) A similar reduction was found in the human CD8^+^ PBMC cell population. *n* = 5–7 biological repeats. [F(1,30) = 6124, *p* < 0.0001, two-way ANOVA]. In all conditions, the vehicle solution had no impact on the doubling rate. (**C**,**F**) rArg-I treatment did not affect murine T-cell survival. *n* = 5 biological repeats. (**I**) Human blood-derived CD4^+^ T cells showed significantly decreased viability when incubated with rArg-I compared to the vehicle when concentrations higher than 100 ng/mL were applied. *n* = 5–7 biological repeats. [F(1,30) = 34.63, *p* < 0.01, two-way ANOVA]. (**L**) Human CD8^+^ PBMCs showed no sign of decreased viability for rArg-I. *n* = 5–7 biological repeats. (**C**,**F**,**I**,**L**) The mean survival of control samples not exposed to vehicle or rArg-I is indicated by the dotted line. The cell viability is indicated as the percentage of FVD eFluor506-negative cells within the CD4^+^ or CD8^+^ T gates, respectively. The vehicle solution had no impact on the cell survival for both murine and human cells. Grey-filled bars are vehicle controls, black-filled bars represent rArg-I-treated cells. Data are shown as mean ± SEM. * *p* < 0.05, ** *p* < 0.01, *** *p* < 0.001, and **** *p* < 0.0001.

We next determined the impact of rArg-I mediated L-arginine depletion on T-cell activation. Primary splenocytes were stimulated in L-arginine containing medium supplemented with different concentrations of rArg-I or vehicle solution. T-cell activation was studied through proliferation analysis (Figure 5). The doubling rates of murine CD4^+^ T cells treated with rArg-I were comparable to controls when the added rArg-I concentrations remained below 100 ng/mL (Figure 5A). Higher concentrations of rArg-I (500 and 1000 ng/mL) halted CD4^+^ T cell proliferation (Figure 5A,B). In line with these findings, murine CD8^+^ T-cell proliferation was also inhibited by rArg-I in a concentration-dependent manner (Figure 5D,E). Similar results were obtained with human PBMCs. Cell proliferation was significantly halted when rArg-I concentrations of 50 ng/mL or higher were added (Figure 5G,H,J,K). The vehicle solution had no impact on T-cell proliferation for both murine and human CD4^+^—and CD8^+^ T cells. Furthermore, the cell viability of murine CD4^+^ and CD8^+^ T cells was not influenced by the used concentrations of rArg-I or the vehicle solution (Figure 5C,F). For the human PBMCs, T-cell survival was only affected in the CD4^+^ subpopulation when cells were incubated with rArg-I concentrations equal to or higher than 250 ng/mL (Figure 5I). Cell survival of the CD8^+^ T cytotoxic cell subpopulation was not significantly altered by adding any of the tested concentrations of the vehicle or rArg-I solution (Figure 5L).

### 3.4. L-Arginine Depletion Reduces NO Production, Neurotoxicity, and the Number of Phagocyte/Axon Contacts

**Figure 6:**
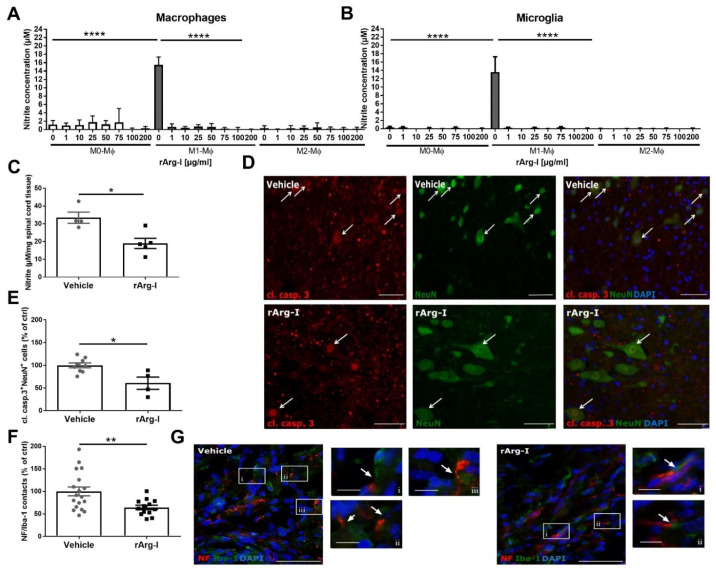
L-arginine depletion through rArg-I supplementation reduces NO production, neurotoxicity, and the number of phagocyte/axon contacts. (**A**) The NO production of bone marrow-derived macrophages exposed to inflammatory stimuli (M1-Mϕ) was significantly attenuated when rArg-I was added to the culture media (added concentrations indicated below graph). As a negative control, non-polarized (M0-Mϕ) and rIL-13 incubated macrophages (M2-Mϕ) were used. *n* = 5–9 biological repeats. [F(23,112) = 26.81, *p* < 0.0001, one-way ANOVA]. (**B**) Griess assay showed similar results on primary microglia; supplementation of rArg-I significantly decreases the nitrite concentration in the presence of inflammatory stimuli. *n* = 4–5 biological repeats. [F(23,83) = 11.1, *p* < 0.0001, one-way ANOVA]. (**C**) The perilesional nitrite concentration in spinal cord homogenates was significantly reduced 7 days post-injury (dpi) in rArg-I-treated animals compared to vehicle controls. *n* = 4–5 mice/group. [31.54 vs. 18.56, *p* = 0.0317, two-tailed Mann–Whitney test]. (**D**,**E**) Histological analysis and representative images of the cleaved caspase 3/NeuN staining of spinal cord tissue from spinal cord injury (SCI) animals treated with vehicle solution or rArg-I, respectively. A marked reduction in the number of cleaved caspase 3^+^ NeuN^+^ double positive cells (white arrows) is observed 28 dpi in rArg-I-treated mice. *n* = 4–9 mice/group. [98.85 vs. 61.84, *p* = 0.0336, two-tailed Mann–Whitney test]. (**F**,**G**) Quantification and representative pictures of perilesional macrophage/microglia and axon contacts 28 dpi. Treatment with rArg-I significantly reduced the number of contacts between Iba1^+^ and NF^+^ cells (white boxed regions). The white boxed regions (**i**–**iii**) in the micrographs are shown in a higher magnification to indicate examples of microglia/macrophage and axon contacts. Data are pooled from two independently performed experiments. *n* = 13–18 mice/group. [100 ± 9.923 vs. 64.32 ± 4.923, *p* = 0.0077, two-tailed unpaired student t-test]. (**D**,**G**) Scale bars represent 50 µm, for the magnified pictures i–iii 10 µm. Data represent mean ± SEM. Cl. Casp. 3: cleaved caspase 3. * *p* < 0.05, ** *p* < 0.01, **** *p* < 0.0001.

Besides the crucial role of L-arginine in T-cell functionality, it is also an important substrate for NO production and the related secondary damage to the spinal cord tissue [47,48]. Therefore, our treatment could provide relief to potential NO-mediated cytotoxicity by reducing L-arginine availability for NOS. Macrophages and microglia are the main NO-producers after SCI [49]. To validate whether rArg-I is able to decrease NO production, nitrite concentrations in the supernatants of macrophage cell cultures were measured. Administration of rArg-I to the culture media significantly decreased the NO production of macrophages when stimulated with pro-inflammatory cytokines (Figure 6A). The decrease of NO production was not the result of reduced NOS expression nor cell death. Macrophage polarization and cell viability were not affected by the presence of rArg-I (Supplementary Figure S5). Similar results were obtained when microglia were exposed to these conditions (Figure 6B). 

A reduction of local NO concentrations is an indirect indication of local L-arginine depletion [43]. To investigate whether systemic L-arginine depletion results in local arginine depletion, we investigated the local in vivo nitrite production in the spinal cord 7 dpi, at the peak of iNOS expression [44]. 

In alignment with the in vitro data, a significant reduction in nitrite concentrations was detected in perilesional spinal cord tissue 7 dpi, confirming the rArg-I mediated effect on NO production in vivo (Figure 6C). 

Furthermore, significantly decreased numbers of cleaved caspase 3^+^ NeuN^+^ double-positive cells were found in the perilesional spinal cord when mice were treated with rArg-I compared to vehicle control (Figure 6D,E). As NO is known to be neurotoxic, the diminished NO production in rArg-I-treated mice could explain the markedly reduced number of apoptotic neurons. In addition, the number of destructive macrophage/microglia—neuron interactions was significantly lower in the treatment group 28 dpi (Figure 6F,G).

## 4. Discussion

Controlling neuroinflammation, especially the processes of inflammation-induced secondary spinal cord damage, is crucial to improve the outcome of SCI patients. Only a few studies so far have investigated the impact of L-arginine depletion in neuroinflammatory pathologies [26,50]. Moreover, limited investigation has been conducted on the effects of L-arginine administration on SCI outcome, showing contradictory results [29,30,31,32]. Tuncer et al. and Yüceer and colleagues reported a beneficial effect of L-arginine administration [30,32]. Tuncer et al. showed improved functional recovery 24 h after ischemia-induced SCI in rats and L-arginine infusion (200 g/kg/min intravenous infusion for 20 min.) [30]. Yüceer et al. found reduced cell damage in the spinal cord 24 h after contusion injury and L-arginine application (concentration not mentioned) [32]. In contrast, Esquivel-Aguilar et al. found a detrimental impact on functional recovery 8 weeks post-injury after 300 mg/kg L-arginine administration and contusion SCI. Moreover, Savas et al. showed an increase of detrimental NO production and NO-mediated cell damage 2 h after ischemia-induced SCI and 100 mg/kg L-arginine administration [29,31]. These conflicting findings might result from differences in the applied L-arginine concentration, the chosen SCI model, and time point for examining the tissue integrity and functional recovery. The beneficial results and high L-arginine concentration applied by Tuncer et al., for example, suggest that high L-arginine concentrations might be necessary to elicit improved results. Here, for the first time, we demonstrate that the systemic administration of rArg-I is beneficial in a well-established mouse SCI model [34,51,52]. Treatment with rArg-I markedly reduced intraspinal T-cell accumulation, elicited acute systemic immunosuppression, alleviated cytotoxic NO production, and improved functional recovery after SCI. Functional recovery is harder to attain in a severe SCI model compared to injury models induced by a weak lesion. Therefore, the observed amelioration of functional recovery is considered statistically and biologically significant as it occurred in a severe T-cut hemisection mouse model. 

L-arginine plays an essential role in wound healing and dural repair [53,54]. Interestingly, our results indicate no effects on lesion size and demyelinated area, yet emphasize the immunomodulatory capacity of L-arginine depletion after SCI. A significant decrease of the number of intraspinal CD4^+^ T cells was noted 28 dpi. Moreover, the systemic (splenic) inflammatory response was alleviated after peripheral rArg-I treatment 4 and 7 dpi. Peripheral inflammation exacerbates SCI by stimulating an excessive inflammatory response which causes damage to initially spared neuronal tissue [55]. As such, the modulation of peripheral inflammation provides neuroprotection in numerous SCI studies [17,56,57]. T-cell activation after SCI occurs first in the spleen. After activation, T cells migrate to the spinal cord and are reactivated (increasing T cell numbers further), contributing to the detrimental inflammatory response. The observed highly significant decrease of CD4^+^ T cell numbers in the spinal cord (*p* < 0.0001) versus the statistically significant but small differences of T cell numbers in the spleen, however, suggest that local alternations may be responsible for improved functional recovery.

L-arginine is an essential regulator of immune cell functioning. rArg-I treatment already resulted in a delayed and ameliorated onset of EAE [26]. T cells, in particular, are highly sensitive to altered L-arginine levels, and arg-1 expression by MDSCs halted T-cell activation [58]. Moreover, research showed that L-arginine availability is necessary for T-cell proliferation and proper T-cell receptor functioning [25,45]. Accordingly, we demonstrate a significant reduction in intraspinal CD4^+^ T cell numbers and splenic Th1, Th17, and Th1/17 subsets 4 dpi and a general decrease of T cell numbers 7 dpi. Five days later (12 dpi), these alterations disappeared, and only an increase of the number of cytotoxic T cells was found.

Although subject to debate and requiring further investigation, several research groups pointed out the detrimental effects of T cell accumulation in the neuropathology after SCI, depending on which T-cell subtype is present at which time point after trauma [12,15,16]. T cells directed against CNS auto-antigens have been reported in both SCI animal models and patients [7]. It is believed that this trauma-induced autoimmunity is caused by an imbalance between activated autoantigen-specific Th cells and Treg cells [7]. Moreover, there is increasing evidence that Th cell responses, in particular Th17 cells, are deleterious in SCI [13,14]. Therefore, it is tempting to connect the detected reduction of Th17 and pro-inflammatory Th1 cells in this study with the improved outcome and/or the reduced number of CD4^+^ T cells in the spinal cord 28 dpi.

To explain the detected reduction in Th1 and Th17 cell numbers in vivo, we tested their activation capacity in the presence or absence of rArg-I in vitro. We observed a concentration-dependent decline of CD4^+^ and CD8^+^ T-cell proliferation. T-cell activation is known to be correlated with an increased L-arginine metabolism, and supplementation of L-arginine can boost human and mouse T-cell proliferation [23,59]. Consistently, our data show that L-arginine starvation, via rArg-I administration halts cellular proliferation, explaining the lowered T cell numbers in the spleen and spinal cord after injury. Though the sample size was in line with previously published studies in the field [23,60,61,62], these data have to be interpreted with care because of the limited number of biological replicates/blood donors (proliferation assay *n* = 5 mice and *n* = 7 human donors). In addition, the ratio of one male versus six female donors may be criticized. However, when the blood sample and corresponding T cells of the male donor were excluded, the same statistical difference (*p* < 0.0001) is obtained for the performed proliferation experiment. A suppressed cyclin D, cdk4, and cdk6 protein expression and transcription lie at the base of the blocked cell proliferation in the absence of L-arginine, impeding cell cycle progression [25]. Moreover, our data indicate that L-arginine depletion via rArg-I did not elicit cell death. This is in line with previous studies [46,63]. Hence, noted changes in the quantity of specific T-cell subtypes and CD4^+^ T cells in the spinal cord of rArg-I-treated mice, likely resulted from decreased proliferation rather than apoptosis or impaired differentiation. Thus, one mechanistic explanation for rArg-I-induced functional recovery may be decreased T-cell mediated neuroinflammation due to reduced T-cell proliferation.

Infiltrating macrophages predominantly originate from the splenic monocyte reservoir with a preferential recruitment of Ly6C^high^ CD11b^high^ inflammatory monocytes to the injured spinal cord [64]. Once infiltrated, they primarily express classically activated (Mca/M1) polarization markers, though discrepancies are reported [64,65].

In our study, the splenic Ly6C^high^ CD11b^high^ monocyte population was significantly reduced in animals treated with rArg-I 4 and 7 dpi. Despite these acute systemic effects, we did not observe significant differences for Iba-1 intensity or polarization status in the spinal cord 28 dpi. This might be explained by the high turnover of the blood-derived monocytes, making it difficult to see differences in cell infiltration/accumulation in the subacute phase later in time [64]. Moreover, our data provide no evidence that rArg-I influences the polarization state of macrophages and microglia in vitro nor in the spinal cord of rArg-I-treated animals 7 dpi (Figure 1 and Supplementary Figure S5). The temporal changes in the number of patrolling and inflammatory splenic monocytes remain to be clarified in further studies.

In the context of spinal cord trauma, NO is one of the known inducers of neuronal cell death [47,66]. The present study indicates a significant reduction of the perilesional NO production 7 dpi when systemic L-arginine depletion was accomplished. Griess assays in vitro on both microglial and macrophage cell cultures confirmed the hampered NO production in the presence of pro-inflammatory stimuli and rArg-I. These findings suggest that the obtained systemic L-arginine depletion via rArg-I results in a perilesional L-arginine deficiency, because reduced local NO concentrations are an indirect indication of local L-arginine depletion [43]. In line with these findings, we also observed a significant decrease of the number of Iba-1^+^ microglia/macrophages and NF^+^ axon contacts 4 weeks after SCI. Activated microglia/macrophage-axon interactions play a direct role in axonal retraction of injured axons and are a hallmark of the injured spinal cord [67]. However, the reduced number of apoptotic neurons might also explain the reduced physical interactions between Iba-1^+^ microglia/macrophages and NF^+^ axons. For the clearance of dying neurons, physical interaction between phagocytes and apoptotic cells is necessary [68]. Since our results indicated less neuronal cell death, less clearance of apoptotic neurons/axons and, therefore, fewer neuron-microglia/macrophage contacts would be necessary.

Together, these findings suggest the following as an additional mechanism of our treatment: rArg-I induces a systemic depletion of L-arginine. This leads to lower local arginine levels, which in turn result in lower NO concentrations, reduced NO-mediated cytotoxicity and reduced neuronal apoptosis. Decreased neuronal death results in less severe lesions and, consequently, to a better functional recovery. It remains to be elucidated why the decreased cleaved caspase 3^+^ NeuN^+^ ratio is associated with an unchanged MBP^-^ area. This might be explained by the unchanged amount of phagocytes (Iba1 intensity), necessary for myelin phagocytosis. Additionally, neuronal survival and de/remyelination are not always closely correlated. In multiple sclerosis, for example, neurons are generally preserved under demyelinating conditions [69].

## 5. Conclusions

In summary, our study shows for the first time the essential role of L-arginine in controlling the systemic inflammatory response after SCI. Two major mechanisms may underly the beneficial effects of rArg-I treatment. Firstly, decreased T-cell mediated neuroinflammation due to reduced T-cell proliferation. Secondly, reduced NO-mediated cytotoxicity and reduced neuronal apoptosis. Our findings suggest that these two processes are crucially involved in secondary damage after SCI and can be therapeutically targeted by systemic rArg-I administration.

## Data Availability

The data that support the findings of this study are available from the corresponding author, upon reasonable request.

## Supplementary Materials

The following are available online at https://www.mdpi.com/article/10.3390/biomedicines10020205/s1, Table S1: Primary antibodies used for immunohistochemistry, Table S2: Secondary antibodies used for immunohistochemistry, Table S3: Primers used for quantitative RT-PCR; Figure S1: Manual gating strategy of murine splenocytes isolated 4–12 days after spinal cord injury, Figure S2: rArg-I influences the splenic immune response 7 days after spinal cord injury, Figure S3: T cytotoxic cell and patrolling monocyte numbers are altered by rArg-I treatment 12 days after trauma, Figure S4: rArg-I treatment leads to a stable serum L-arginine depletion over time, Figure S5: rArg-I addition does not lead to cell death or macrophage polarization in vitro.
