# Supplementary material for: L-Arginine Depletion Improves Spinal Cord Injury via Immunomodulation and Nitric Oxide Reduction
Source: Biomedicines. 2022 Jan 18;10(2):205. doi: 10.3390/biomedicines10020205 (PMC8869469; doi:10.3390/biomedicines10020205)
Supplement: Supplementary file 1 [file biomedicines-10-00205-s001.zip › biomedicines-1426197-supplementary.pdf]

Supplementary Table S1: Primary antibodies used for immunohistochemistry.

Antibody	Antibody type	Target	Source	Dilution
GFAP	Mouse (monoclonal)	Glial fibrillary acidic protein	G3893, Sigma-Aldrich	1/500
MBP	Rat (monoclonal)	Myelin basic protein	MAB386, Merck Millipore	1/250
Iba-1	Rabbit (polyclonal)	Ionized calcium-binding adaptor molecule	019-19741, Wako	1/350
Iba-1	Goat (polyclonal)	Ionized calcium-binding adaptor molecule	NB100-1028, Novus Biologicals	1/250
CD4	Rat (monoclonal)	Cluster of differentiation 4	553043, BD Biosciences	1/250
Arg-1	Goat (polyclonal)	Arginase-1	sc-18354, Santa Cruz	1/50
MHCII	Rat (monoclonal)	Major histocompatibility complex II	sc-59322, Santa Cruz	1/200
cl. casp. 3	Rabbit (polyclonal)	Cleaved caspase 3	9661, Bioké	1/100
NeuN	Mouse (monoclonal)	Neuronal nuclei	MAB377, Merck Millipore	1/1000
NF	Rabbit (monoclonal)	Neurofilament	MA5-14981, Thermo Fisher Scientific	1/100

Supplementary Table S2: Secondary antibodies used for immunohistochemistry.

Antibody type	Label	Source	Dilution
Goat anti-mouse IgG (Polyclonal)	Alexa Fluor 568	A11004, Invitrogen	1/250
Goat anti-rat IgG (Polyclonal)	Alexa Fluor 488	A11006, Invitrogen	1/250
Donkey anti-goat IgG (Polyclonal)	Alexa Fluor 555	A21432, Invitrogen	1/250
Goat anti-mouse IgG (Polyclonal)	Alexa Fluor 488	A21121, Invitrogen	1/400
Donkey anti-goat IgG (Polyclonal)	Alexa Fluor 488	A11055, Invitrogen	1/400
Donkey anti-rabbit IgG (Polyclonal)	Alexa Fluor 555	A31572, Invitrogen	1/400

Supplementary Table S3: Primers used for quantitative RT-PCR.

Gene	Target	Forward primer (5'-3')	Reverse primer (5'-3')
CD45	Cluster of differentiation 45	ACCCAGTGATGAACTGAGCA	TTGGGGGTGTGGATTCAGTG
CD3	Cluster of differentiation 3	AACACGTACTTGTACCTGAAAGCTC	GATGATTATGGCTACTGCTGTCA
CD8	Cluster of differentiation 8	GCTACCACAGGAGCCGAAA	TCCTGGCGGTGCCATTTTAC
CD4	Cluster of differentiation 4	GAGAGTCAGCGGAGTTCTC	CTCACAGGTCAAAGTATTGTT G
FOXP3	Forkhead box P3	CGGGTACACCCAGGAAAGAC	ATCTGCTTGGCAGTGCTTGA

TNFα	Tumor necrosis factor α	GTCCCCAAAGGGATGAGAAGT	TTTGCTACGACGTGGGCTAC
IFNγ	Interferon γ	TGAGGTCAACAACCCACAGGT	GACTCCTTTTCCGCTTCCTGAG
Tbet	T-box gene	CCACCTGTTGTGGTCCAAGT	AAGCAAGGACGGCGAATGTT
RORγT	Retinoic acid-related orphan receptor γ t	GTGGAGTTTGCCAAGCGGCTTT	CCTGCACATTCTGACTAGGACG
TFGβ	Transforming growth factor β	GGGCTACCATGCCAACTTCTG	GAGGGCAAGGACCTTGCTGTA
CYPA	Cyclophilin A	GCGTCTCCTTCGAGCTGTT	AAGTCACCACCCTGGCA
GAPDH	Glyceraldehyde-3-Phosphate Dehydrogenase	GGCCTTCCGTGTTCCTAC	TGTCATCATATCTGGCAGGTT
HMBS	Hydroxymethylbilane synthase	GATGGGCAACTGTACCTGACTG	CTGGGCTCCTCTTGGAATG
B-actin	B-actin	GGCTGTATTCCCCTCCATCG	CAGTTGGTAACAATGCCA

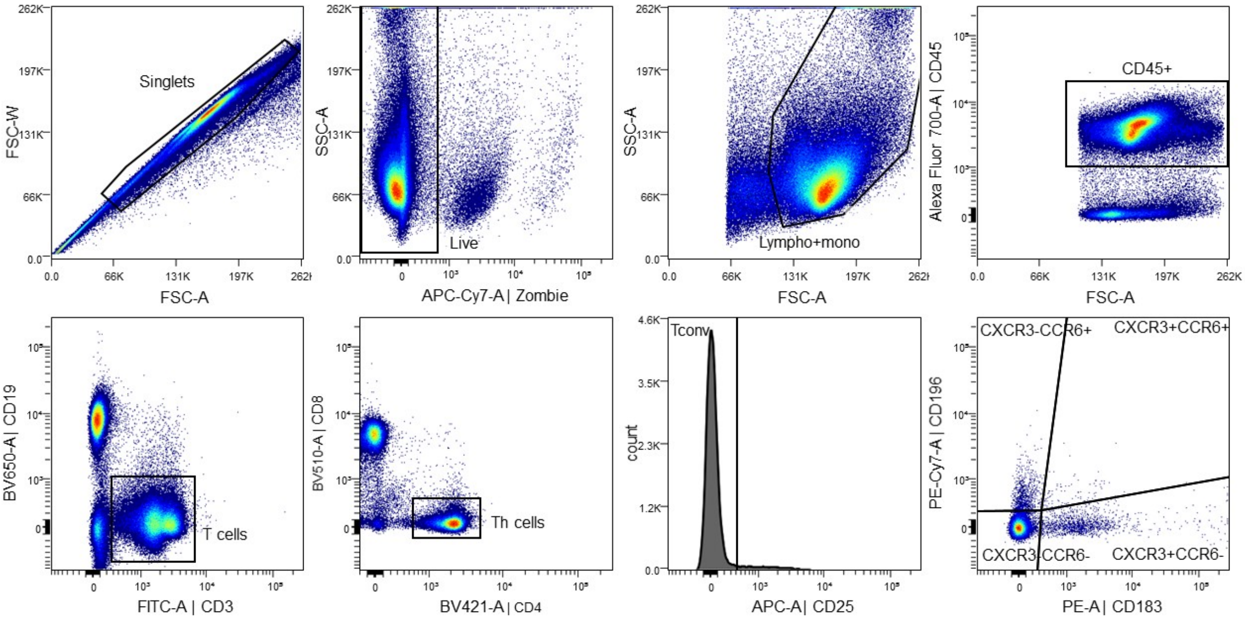

Supplementary Figure S1: Manual gating strategy of murine splenocytes isolated 4 - 12 days after spinal cord injury. Manual gating occurred by a blinded researcher. After gating for singlets and live cells, the lymphocyte gate was formed. Next, T cells and T cell subpopulations were identified.

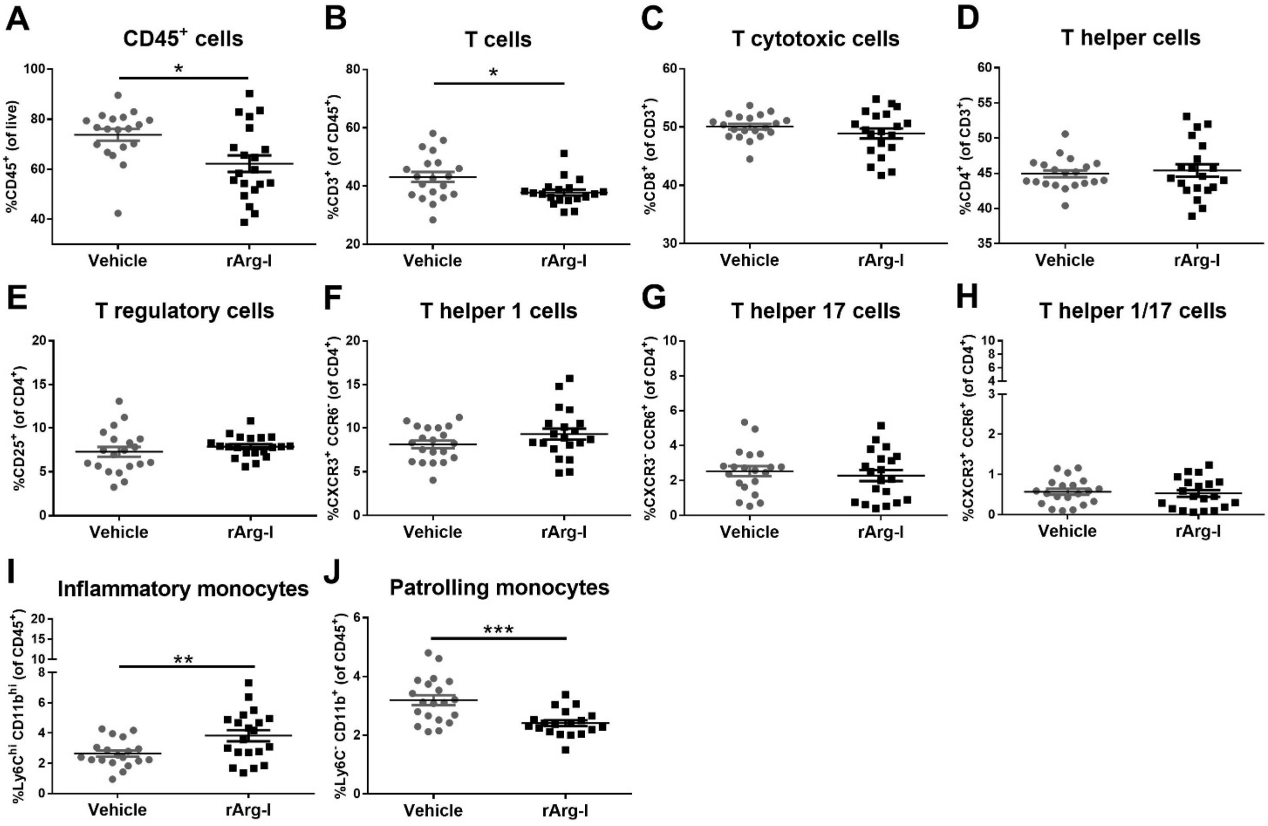

Supplementary Figure S2: rArg-I influences the splenic immune response 7 days after spinal cord injury. Flow cytometry analysis on splenocytes derived from vehicle— or rArg-I-treated mice revealed significant alternations 7 dpi. The number of (**A**) $CD45^+$ leukocytes and (**B**) $CD3^+$ T cells was significantly decreased. n = 19-20 mice/group. [76.2 vs. 59.1 and 42.95 vs. 37.3 respectively, $p = 0.0128$ and $p = 0.0204$, two-tailed Mann Whitney test]. (**C-H**) No significant differences were found in the different T-cell subsets. n = 19-20 mice/group. (**I**) The number of inflammatory splenic monocytes rose significantly 7 dpi. n = 19-20 mice/group. [2.65 ± 0.21 vs. 3.83 ± 0.37, $p = 0.009$, two-tailed unpaired student t test]. (**J**) On the contrary, the number of patrolling monocytes was significantly decreased. n = 19-20 mice/group. [3.19 ± 0.17 vs. 2.41 ± 0.10, $p = 0.0004$, two-tailed unpaired student t test]. Data are represented as mean ± SEM. *$p < 0.05$, **$p < 0.01$, ***$p < 0.001$.

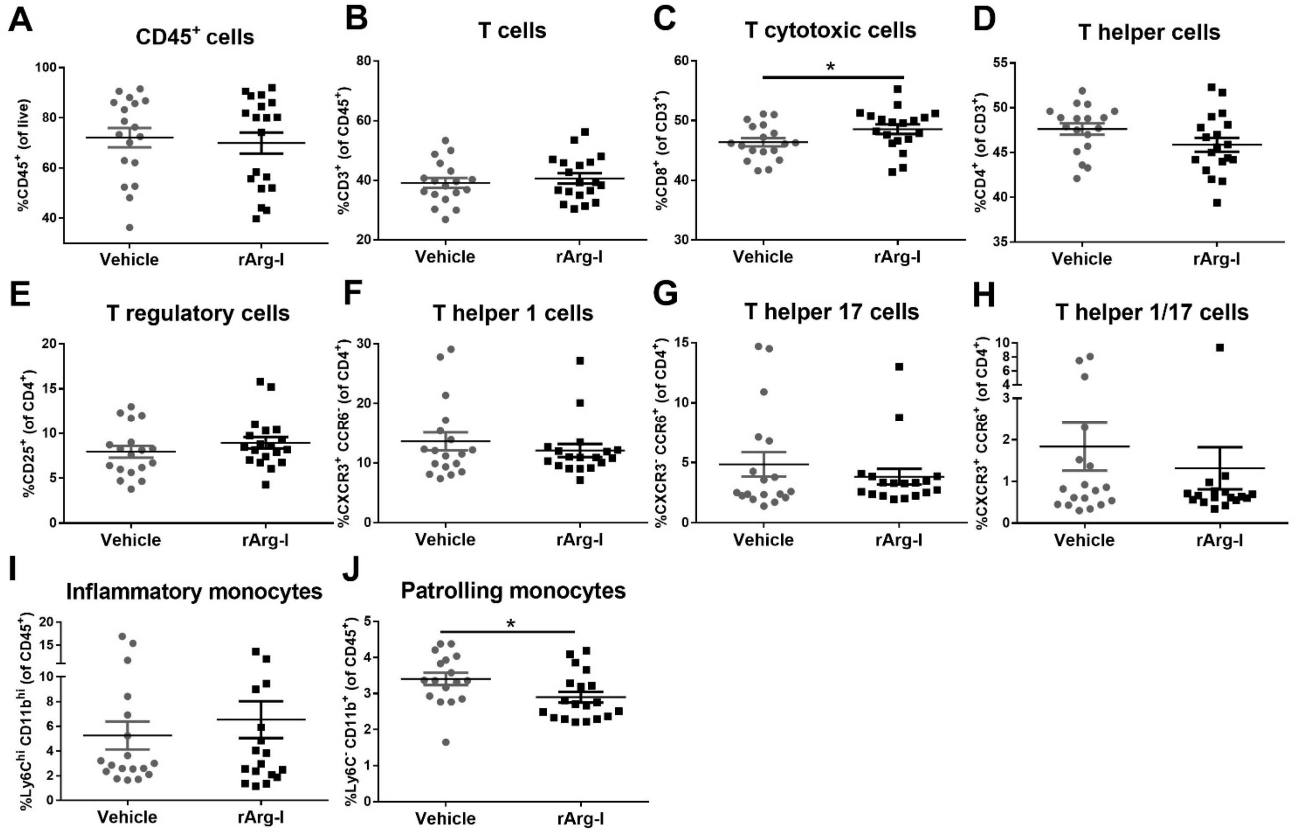

Supplementary Figure S3: T cytotoxic cell and patrolling monocyte numbers are altered by rArg-I treatment 12 days after trauma. (**A-B**) The number of $CD45^+$ leukocytes and $CD3^+$ T cells was not altered by repetitive rArg-I injections. $n = 18\text{-}19$ mice/group. (**C**) Flow cytometry showed a significant increase in the T cytotoxic cell population. $n = 18\text{-}19$ mice/group. [46.38 ± 0.69 vs. 48.56 ± 0.79, $p = 0.0472$, two-tailed unpaired student t-test]. (**D-H**) No significant changes in the number of $CD4^+$ T cell subpopulations were observed at 12 dpi. $n = 18\text{-}19$ mice/group. (**I-J**) Of the splenic monocyte populations, the number of patrolling monocytes significantly decreased while the inflammatory monocyte population remained unaltered. $n = 17\text{-}19$ mice/group. [3.41 ± 0.17 vs. 2.9 ± 0.15, $p = 0.0327$, two-tailed unpaired student t-test]. Data are shown as mean ± SEM. $^*p < 0.05$.

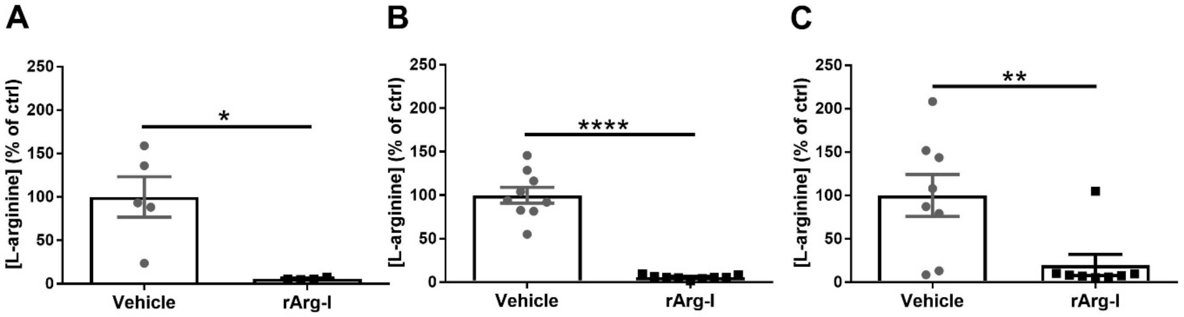

Supplementary Figure S4: rArg-I treatment leads to a stable serum L-arginine depletion over time. (**A**) L-arginine ELISA of serum samples obtained 4 days post-injury (dpi). Treatment of rArg-I significantly reduced the L-arginine levels. n = 4-5 mice/group. [93.29 vs. 5.523, $p = 0.0159$, two-tailed Mann Whitney test]. (**B**) Serum L-arginine concentrations are markedly decreased 7 dpi in rArg-I-treated animals. n = 9 mice/group. [100 ± 9.102 vs. 5.736 ± 0.7565, $p < 0.0001$, two-tailed unpaired student t test]. (**C**) Spinal cord injured mice treated with rArg-I show significantly reduced L-arginine serum concentrations 12 dpi. n = 8 mice/group. [97.65 vs. 7.931, $p = 0.0047$, two-tailed Mann Whitney test]. Data represent mean ± SEM, percentage of vehicle group. *$p < 0.05$, **$p < 0.01$,****$p < 0.0001$.

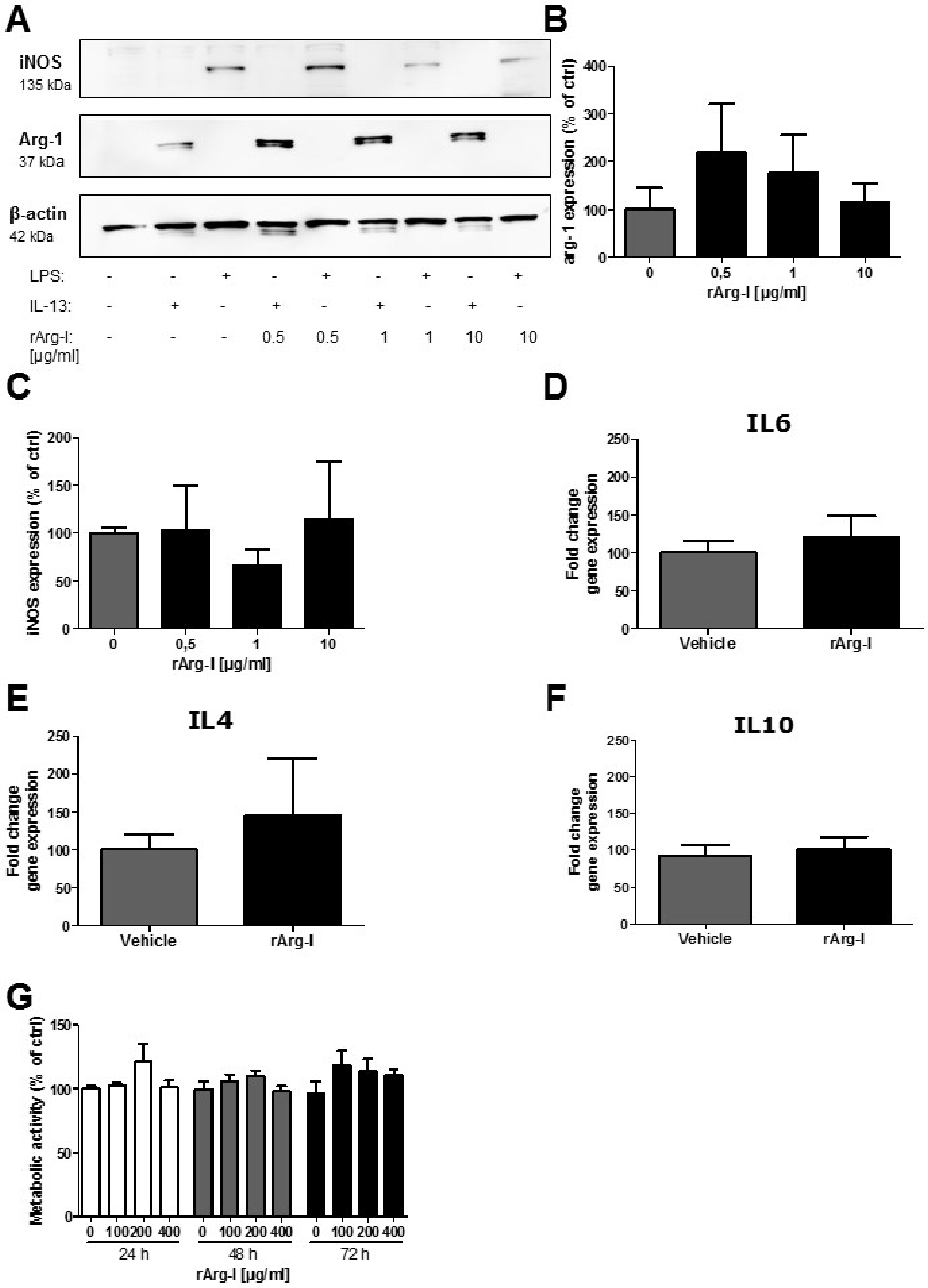

Supplementary Figure S5: rArg-I addition does not lead to cell death or macrophage polarization.

(**A**) Representative western blot of macrophages incubated with LPS or rIL-13 and different concentrations of rArg-I. (**B**) Quantification of arg-1 expression of rIL-13-stimulated macrophages. rArg-I addition did not alter the arg-1 expression. *n* = 5 biological repeats. (**C**) Quantification of iNOS

expression of macrophages incubated with LPS. rArg-I did not affect the iNOS expression when rArg-I was added to the culture medium. *n* = 4 biological repeats. (**D-F**) Quantification of relative mRNA levels in the spinal cord 7 dpi for *IL6, IL4* and *IL10*. No difference was observed between the vehicle and rArg-I treated group. *n* = 17-20 mice/group for *IL6* and *IL10* and *n* = 3-4 mice/group for *IL4*. (**G**) The addition of rArg-I did not alter the macrophage viability *in vitro* over time. MTT showed no significant difference from 24 h to 72 h. Added rArg-I concentrations are indicated in the graphs. Data represent mean ± SEM.
